# Safety-Specific Passive-Avoidant Leadership and Safety Compliance among Chinese Steel Workers: The Moderating Role of Safety Moral Belief and Organizational Size

**DOI:** 10.3390/ijerph18052700

**Published:** 2021-03-08

**Authors:** Lin Liu, Qiang Mei, Lixin Jiang, Jinnan Wu, Suxia Liu, Meng Wang

**Affiliations:** 1 School of Management, Jiangsu University, Zhenjiang 212013, China; liulinahut@aliyun.com (L.L.); qmei@ujs.edu.cn (Q.M.); liusuxia@ujs.edu.cn (S.L.); 2 Key Laboratory of Multidisciplinary Management and Control of Complex Systems of Anhui Higher Education Institutes, Anhui University of Technology, Ma’anshan 243032, China; 3 School of Psychology, University of Auckland, Auckland 1010, New Zealand; l.jiang@auckland.ac.nz; 4 School of Business, Anhui University of Technology, Ma’anshan 243032, China; wangmeng0806@ahut.edu.cn

**Keywords:** passive-avoidant leadership, safety-specific leader reward omission, safety-specific leader punishment omission, safety moral beliefs, safety compliance, steel workers

## Abstract

Despite the documented relationship between active-approaching leadership behaviors and workplace safety, few studies have addressed whether and when passive-avoidant leadership affects safety behavior. This study examined the relationship between two types of safety-specific passive-avoidant leadership, i.e., safety-specific leader reward omission (SLRO) and safety-specific leader punishment omission (SLPO), and safety compliance, as well as the moderating effects of an individual difference (safety moral belief) and an organizational difference (organizational size) in these relationships. These predictions were tested on a sample of 704 steel workers in China. The results showed that, although both SLRO and SLPO are negatively related to safety compliance, SLPO demonstrated a greater effect than SLRO. Moreover, we found that steel workers with high levels of safety moral belief were more resistant to the negative effects of SLRO and SLPO on safety compliance. Although steel workers in large enterprises were more resistant to the negative effects of SLPO than those in small enterprises, the SLRO-compliance relationship is not contingent upon organizational size. The current study enriched the safety leadership literature by demonstrating the detrimental and relative effects of two types of safety-specific passive-avoidant leadership on safety compliance and by identifying two boundary conditions that can buffer these relationships among steel workers.

## 1. Introduction

Injuries and deaths resulting from workplace accidents have always been one of the most costly issues worldwide [1,2,3]. Several studies have established that unsafe acts and conditions or a continuous violations of safety regulations are elements that often lead to incidents, injuries, near-misses or disasters [4,5,6,7]. In particular, steel manufacturing industry is recognized as one of the highly hazardous industries due to its unique nature of the job [2,8]. For example, steel workers are often exposed to high temperature, high dust, high noise, toxic gas and explosive, dangerous sources, which more likely lead to group incidents, injuries and burns than other manufacturing industries [8,9,10]. Thus, to reduce accidents and injuries, it is essential for steel workers to increase safety compliance behaviors [2,9,11,12].

Safety compliance is defined as “the core activities that individuals need to carry out to maintain workplace safety” [13]. A great deal of evidence confirms that safety compliance is associated with fewer accidents and injuries [12,14,15]. Therefore, it is important to improve our understanding of the factors that might influence individual safety compliance. In three meta-analysis studies by Christian et al. [16], Clarke [17] and Nahrgang, Morgeson and Hofmann [5], leadership was categorized as one important distal situation-related antecedent of safety compliance. Recently, interest in safety leadership as an antecedent of safety behaviors has increased, with the majority of literature confirming the influence of active-approaching transformational leadership on followers’ safety compliance [18,19,20,21]. Active-approaching transactional leadership (i.e., leader contingent reward and punishment) has also been found to be an effective way to improve followers’ safety compliance [22,23,24]. While it is understandable to focus on active-approaching forms of safety leadership, not all leadership is positive. Indeed, most followers are likely to experience passive-avoidant (e.g., laissez-faire leadership) rather than active-approaching leadership during their working life [25], because more leaders have not shown to be actively involved in improving workplace safety performance [26]. Actually, the absence of leadership (e.g., leader reward omission and leader punishment omission) is nearly as important as the presence of leadership (e.g., transformational leadership and transactional leadership) [27]. However, compared to the examinations on active-approaching leadership forms, far fewer attempts have been made to study the impact of passive-avoidant leadership [28,29] in general and in the occupational safety context in particular [23,30,31].

Since an early study by Kelloway, Mullen and Francis [26], who found that safety specific passive-avoidant leadership leads to safety events and subsequent injuries through decreasing safety consciousness and safety climate, Mullen, Kelloway and Teed [30]; Grill, Pousette, Nielsen, Grytnes and Törner [22] and Grill, Nielsen, Grytnes, Pousette and Törner [23] established that passive-avoidant leadership negatively relates to safety outcomes with the mediation role of psychological safety climate [31]. Despite these exceptional studies, we noticed that these studies use a relative generalized measure of laissez-faire leadership [32], which may prevent us from getting a more nuanced understanding of the predictive and relative power of different types of safety-specific passive-avoidant leadership on follower safety compliance [33,34,35]. More importantly, these studies do not tell us when passive-avoidant leadership behaviors may have stronger or weaker effects [36] on follower safety compliance. Further, more studies are needed to examine the generalizability of previous research findings with workers from different industries (e.g., steel industry).

To narrow these gaps in the existing literature, the current study aims to examine the effects of two forms of safety-specific passive-avoidant leadership (safety-specific leader reward omission and punishment omission) on follower safety compliance behavior and examine how one organization characteristic (organizational size) and one individual difference (safety moral belief) moderate these relationships among 704 steel workers in China. Specifically, our first goal is to contextualize the general measures of leader reward omission (leader nonreinforcement of subordinate good performance) and punishment omission (leader nonreinforcement of subordinate poor performance) [37] to the specific domain of occupational safety [33,38,39,40] and compare and contrast the effect of two safety-specific leader omissions, reward omission and punishment omission, on followers’ safety compliance behavior. We focus on safety-specific leader reward and punishment omission, because they explicitly capture leaders’ nonreinforcements of followers’ good or poor safety performance and have been found to negatively associate with follower performance [37]. Second, we examine whether two moderators, organizational size and safety moral belief, interact with safety-specific leader reward and punishment omission to shape followers’ safety compliance behavior. This study focuses on organizational size and safety moral beliefs as moderators that are potentially related to safety behavior [41,42,43]. This is because individuals’ compliance decision can be understood as a moral conflict [44], and moral disengagement has been found to buffer the relationship between passive-avoidant leadership and safety non-compliance [36]. Moreover, different size of organizations face different levels of challenges and barriers in managing safety due to differentiated safety investments [43,45]. We draw on the contextual or contingency leadership literature [46,47,48] to build our hypotheses.

The remainder of the introduction describes the theoretical foundation underlying this study and the model and hypotheses proposed.

### 1.1. Safety-Specific Passive-Avoidant Leadership and Safety Compliance

Given that the omission of leadership may be just as important as its commission, Hinkin and Schriesheim [37] introduced the concepts of leader reward omission and punishment omission, representing two types of interrelated concretized passive-avoidant leadership behaviors. According to Hinkin and Schriesheim [37], leader reward omission is defined as leader nonreinforcement of what a follower perceives to be his or her good performance, while leader punishment omission is defined as leaders’ nonreinforcement of what a follower perceives to be his or her poor performance. Hence, different from low level of contingent reward or punishment, leader reward omission or punishment omission means that leader ignores followers’ desirable or undesirable behaviors and makes no positive or negative responses. Unlike prior research focusing on relationships between leadership behaviors and safety outcomes without contextualizing leadership measures to be safety specific [18,20,23,26,31], the present study contextualizes the operationalization of passive-avoidant leadership to be safety-specific, i.e., safety-specific leader reward omission (SLRO) and safety-specific leader punishment omission (SLPO). Both scales reflect that leaders do not engage in motivating and promoting followers’ safety behaviors [4,49].

Passive-avoidant leadership, identified as a form of laissez-faire leadership, shows passive indifference about tasks and workers, ignores worker needs and ignores problems, and has been described as the absence of effective leadership [50]. Thus, passive-avoidant leadership is generally accounted to be the least effective style [51]. Petrock [52] argued that nonresponse to poor subordinate performance may do nothing to elicit the desired behavior. Zohar [53] implied that passive leadership provides little-to-no concern for the well-being of followers with respect to safety and, if continued, will result in unproductive safety initiatives, diminished safety climate perceptions and diminished safety outcomes. Mullen and Kelloway [19] found that followers’ intention to compliance with safety regulations and operating procedures decreases when leaders fail to actively promote safe behaviors and practices. More recent studies further suggested that passive-avoidant leadership practices negatively influence followers’ safety outcomes by encouraging carelessness and unsafe work behavior [22,23,54]. Consistent with these studies, we similarly predict that safety-specific leader reward and punishment omission, as two forms of passive-avoidant leadership in the safety-critical context, will be negatively associated with followers’ safety compliance behavior.

**Hypothesis** **(H1).** *SLRO is negatively associated with safety compliance behavior.*

**Hypothesis** **(H2).** *SLPO is negatively associated with safety compliance behavior.*

Considering that steel workers are faced with a variety of safety risks due to the nature of their work [8], steel enterprises all over the world, including China, have formulated strict safety regulations and operating procedures [3]. Therefore, to improve their occupational health and reduce the cost of enterprise accidents, steel workers are expected and required to abide by these rules and regulations in their daily work [8,55]. The outcomes of ignoring safety noncompliance or violation behaviors is different than overlooking safety compliance behavior [56]. Safety compliance behaviors may be typically less comfortable, convenient or efficient than safety noncompliance or violation behaviors [56]. If someone ignores or even violates safety rules without negative feedbacks or punishments from leaders, they will perceive strong organizational injustice and role ambiguity [37,57], as well as low perceived cost of safety violations [58], which will lead to their undesirable attitude and behavior of safety compliance in the future. Hence, we predict that safety-specific leader punishment omission will have a greater negative impact on followers’ safety compliance than safety-specific leader reward omission.

**Hypothesis** **(H3).** *SLPO has a larger negative impact on safety compliance behavior than SLRO.*

### 1.2. Moderating Role of Safety Moral Belief

Moral beliefs are inculcated perceptions or viewpoints that inhibit individuals from engaging in misconduct [59], and are considered to be motivated intrinsically [60]. Moral beliefs are relevant to the context of workplace safety because choices regarding safety compliance behavior generally involve a moral component, the decision to ignore or even violate safety rules can be understood as a moral conflict [44]. Previous studies have shown that passive-avoidant leadership are related to follower safety outcomes; however, to the authors’ best knowledge, it is unclear whether safety moral beliefs interact with passive-avoidant leadership to predict safety outcomes. Despite a recent study by Olsen, Hetland, Matthiesen, Hoprekstad, Espevik and Bakker [36] that investigated how dispositional moral disengagement buffers the relationship between passive-avoidant leadership and safety non-compliance, no study has yet to examine the interaction between passive-avoidant leadership (i.e., SLRO and SLPO) and safety moral beliefs on follower safety compliance behaviors.

Moral beliefs studies have consistently shown that moral proscriptions act as a deterrent for many forms of criminal or deviant behaviors [61,62,63,64]. Some acts are not committed because it is believed to be morally incorrect [64]. Bandura et al. [65] argued in social cognitive theory that one’s moral and immoral behaviors are a function of the self-regulatory mechanism of self-monitoring and self-reactions. Specifically, when moral beliefs are strongly held by individuals, formal rewards and punishments are then not needed [66]. Therefore, Bachman, Paternoster and Ward [66] considered moral beliefs to be a more important form of social control than deterrence-oriented perceptions, and moral inhibitions alone were effective constraints in some situations. Further, moral beliefs act as a buffer in explaining the relationship between predictors and criminal/deviant behaviors. For example, Bachman, Paternoster and Ward [66] found that the threat of sanctions was not a deterrent for those with high moral beliefs. Consistent with Bachman, Paternoster and Ward [66], Schoepfer and Piquero [62] suggested that low self-control relates to crime only under conditions of high moral beliefs. Given the fact that existing safety leadership research focusing on the leadership style or behavior fails to account for the influence of followers’ individual differences, this study, following Bandura, Barbaranelli, Caprara and Pastorelli [65], Bachman, Paternoster and Ward [66] and Schoepfer and Piquero [62], examines the moderating role of safety moral beliefs on the effect of safety-specific leader reward and punishment omission on employee safety compliance. When safety-specific moral beliefs are strongly held by steel workers, they favor to comply with safety rules even if safety-specific leader reward and punishment are omission, because they evaluate non-compliance or violation as morally wrong [63] and feel obligated to comply with safety rules [62]. Thus, we expect safety-specific leader reward and punishment omission to have larger (less) effect on safety compliance behavior under conditions of low (high) safety moral beliefs.

**Hypothesis** **(H4).** *Safety moral belief will moderate the relationship between SLRO and safety compliance behavior: An individual’s perception of SLRO will be more strongly associated with safety compliance behavior when safety moral belief is low than when it is high.*

**Hypothesis** **(H5).** *Safety moral belief will moderate the relationship between SLPO and safety compliance behavior: An individual’s perception of SLPO will be more strongly associated with safety compliance behavior when safety moral belief is low than when it is high.*

### 1.3. Moderating Role of Organizational Size

The contextual leadership literature has well-documented that leadership effectiveness depends not only on task, physical and temporal context but, also, on social context such as organizational characteristics [46,48]. Organizational size describes the size of the enterprise in terms of the number of employees, which influences many factors, such as provision of economic resources and time, organic and organized approach to safety and so on [41]. Previous safety behavior studies primarily focused on either large enterprises, small enterprises or as a whole [8,41,42,67,68]. Findings from these research suggest that safety performance of small businesses is poorer than that of larger ones [41,69,70], because they have higher exposure to occupational hazards [42] and, further, face distinct challenges and barriers in managing safety due to fewer resources [43,45]. Further, Yin and Yang [10] indicated that the death rate per million-ton of steel in small enterprises (with less than 300 employees) is 100–200% higher than that in large enterprises (with more than 1000 employees). Despite these weaknesses in safety promotion and management among small enterprises, only few studies have examined the differences in safety risk, climate and injuries between small and large enterprises [42,45]. With regard to the relationship between safety leadership and safety compliance, to date, no studies have explicitly examined the moderating effect of organizational size on the relationship between leadership style, especially passive-avoidant leadership and followers’ safety compliance.

In line with the contextual leadership research, this study aims to expand our understanding of safety leadership effectiveness by examining how safety-specific leader reward and punishment omission relates to followers’ safety compliance behavior at different organizational sizes. Specifically, compared with large enterprises, small steel enterprises may not manage workplace safety effectively and may be unaware of their responsibilities under the occupational health and safety law [71]. Their investment in improving occupational health and safety at the workplace may be insufficient due to limited financial resources and invisible short-term benefits [69], which make it difficult or impossible to follow the rules [72]. Moreover, organization process is also related to organizational size [73]. Small enterprises tend to show several deficiencies in organizational process relevant to safety, such as safety training, safety communication and safety management systems [74]. Accordingly, when safety-specific leader reward and punishment are omission, low levels of safety compliance will be more acceptable to workers in small steel enterprises. Moreover, workers in small steel enterprise have greater time pressure than those in large enterprises, which may further lead to decreased motivation to comply with safety rules and regulations [75,76]. Contrary to small enterprises, workers in large ones are provided with more safety-specific organizational support as a result of sufficient safety-specific financial and equipment resources, strict safety-specific operation procedures and complete safety management systems, which will motivate them to behave more safely [7]. Hence, even if there is a lack of safety-specific leader rewards and punishments, workers in large steel enterprises tend to comply with safety rules and regulations because of sufficient resources and safety training. Based on the above discussion, we hypothesized that organizational size buffers the relationship between safety-specific leader reward and punishment omission and followers’ safety compliance behavior.

**Hypothesis** **(H6).** *Organizational size will moderate the relationship between SLRO and safety compliance behavior: SLRO will be more strongly associated with safety compliance behavior when organizational size is small than when it is large.*

**Hypothesis** **(H7).** *Organizational size will moderate the relationship between SLPO and safety compliance behavior: SLPO will be more strongly associated with safety compliance behavior when organizational size is small than when it is large.*

**Figure 1:**
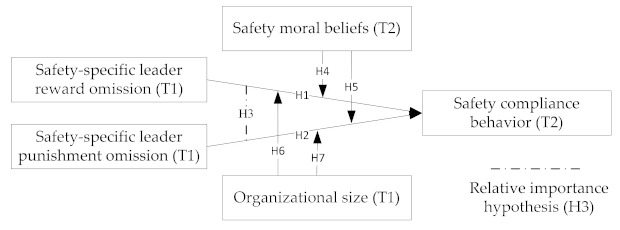
Relations among research variables according to hypotheses. Note: T1 and T2 represent the first and second wave data collection, respectively.

On the basis of the above hypotheses, Figure 1 demonstrates the relations among the dimensions.

## 2. Materials and Methods

### 2.1. Participants and Procedure

A two-wave, self-reported survey with a two-week time lag in between was used to test our hypotheses. We focused on front-line workers in steel enterprises located in Anhui, one of China’s largest iron and steel producing provinces. Although the participants were not selected from the entire China, they were heterogeneous and representative in terms of the number of the employees. Moreover, our recruitment method ensured that participants match the research context of workplace safety of this study.

At the baseline assessment (T1) of the two-wave survey, participants were asked to report the frequency with which their superior engaged in safety-specific leader reward omission and punishment omission and answer demographic questions as the last part of the questionnaire recommended by Brondino et al. [77]. The second wave of the data (T2) was collected two weeks later. During this stage, participants were asked to report their own perceptions of safety moral beliefs and actual safety compliance behaviors in the past two weeks. To reduce potential effect of socially desirable responding, we administered the questionnaire on an online platform due to its advantage in higher anonymity and reliability in collecting sensitive information [78,79].

Considering the situation of COVID-19 epidemic, we conducted an online survey to collect data. With the help from the Emergency Management Bureau, research assistants distributed questionnaire to participants with a quick response (QR) code through WeChat, one of the most popular instant messaging and social interaction application in China and all over the world [80]. Prior to starting the survey, participants were informed that all data would be protected and that only aggregate results would be used for statistical analyses. Following this, they were asked to take about five minutes to understand the purpose and instructions of this survey. All participants answered the questionnaire at the end of their week days.

The data collection process lasted about three weeks in April 2020. In the first stage, 1250 questionnaires were initially distributed and 1028 filled out and returned, including 32 invalid questionnaires, and 996 were retained. In the second stage, the T2 survey was only distributed to those who responded to the T1 survey by matching the username (when users register in this survey platform, a unique username will be automatically assigned for him/her) and IP address, and 742 questionnaires were collected. After eliminating 38 invalid questionnaires, a total of 704 usable samples were retained for data analyses (56.3 per cent usable response rate). Following Ye et al. [81], an independent sample *t*-test was performed to compare the first 10 per cent and the last 10 per cent of respondents to test nonresponse bias. No significant difference was found between two groups across demographic and focal variables in our hypotheses.

Of 704 responses, 61.8 per cent were men. The participants were evenly distributed among different age groups: between 20 and 30 years (29%), between 31 and 40 years (39.3%) between 41 and 50 years (24.6%), and larger than 50 years (7.1%). The most common educational level was university or above (42.8%), followed by high school or technical secondary school (30.8%) and junior high school or below (26.4%). More than half (52.7%) reported earning monthly income between 3000 and 5000 Yuan followed by less than 3000 Yuan (25%), between 5001 and 7000 Yuan (14.3%), and more than 7000 Yuan (8%). Most of the samples (84.8%) signed formal employment contracts with their employers. Working years included less than 4 years (32.8%), between 4 and 10 years (37.4%) and more than 10 years (29.8%). Finally, the participants came from steel enterprises of different scales, including enterprises with an employee size of more than 5000 (4.5%), between 1001 and 5000 (45.2%), between 501 and 1000 (14.5%), between 301 and 500 (20.5%), between 51 and 300 (5.1%) and less than 50 (10.2%). However, we did not collect information on the employees’ organization to preserve their anonymity.

### 2.2. Measures

In accordance with the recommendations of previous studies on measure contextualization [33,35,38,82,83], we tagged widely used multi-item scales with safety context to measure safety-specific leader reward and punishment omission, safety moral beliefs and safety compliance in this study. All items are shown in Table 1 in detail.

Safety-specific leader reward and punishment omission. The scale includes two dimensions of SLRO and SLPO, which were contextually constructed and measured based on the study of Hinkin and Schriesheim [37]. SLRO were assessed with six items, while SLPO were measured with five items. Participants rated how often their superior engaged in reward and punishment omission behaviors on a 5-point Likert-type scale (1 = never and 5 = always).

Safety compliance behavior. Safety compliance behavior reflects the degree to which steel workers comply with safety rules and procedures. It was measured as the criterion using a five-item scale where three items were adopted from Neal and Griffin [13], and the other two were incorporated from Mei et al. [84] to capture the safety practices among firms in China. We match predictor and criterion specificity to increase the predictive validity of self-report measures of safety-specific leader reward omission and punishment omission [33,82]. All items were evaluated on a 5-point Likert-type scale (1 = strongly disagree and 5 = strongly agree).

Safety moral beliefs. The items for assessing safety moral beliefs were contextualized and adapted from moral belief items validated by Vance and Siponen [61]. It was measured by a three-item scale. The first two items were evaluated on a 5-point Likert-type scale (1 = strongly disagree and 5 = strongly agree), while the last one was evaluated on a 5-point Likert-type scale (1 = slightly and 5 = seriously).

Control variables. In order to control for alternative explanations of our results, we controlled for the additional social and demographic variables such as gender, age, education, income, employment type and working years [76,85,86,87].

### 2.3. Statistical Analysis

We used multiple regression analysis to test Hypothesis 1 and Hypothesis 2, i.e., the main effect of SLRO and SLPO on safety compliance behavior. Hypothesis 3 (the differential effect between SLRO and SLPO on safety compliance behavior) was evaluated by computing the significance level and BC (Biased corrected) 95% confidence interval of the difference between two effect sizes with Mplus 7.0. For the contingent hypotheses (Hypothesis 4 to Hypothesis 7), in which safety moral belief and organizational size were used as moderators, moderated regression analysis was employed [88].

## 3. Results

### 3.1. Common Method Bias Test

We used Harman’s one-factor test to assess common method bias (CMB) [89]. The factor analysis based on principal components extracted common factors and performed orthogonal rotation with the varimax procedure, finally generating four principal components (with an eigenvalue greater than 1). The first principal component explained 35.9% of the variance, indicating that CMB is not a likely contaminant of our results. We also used confirmatory factor analysis (CFA) with Mplus 7.0 to confirm the result above as recommended by Slater et al. [90]. The CFA results show that the fit indices of the four-factor model, χ^2^ = 815.0, SRMR (Standardized Root Mean-square Residual) = 0.028, CFI (Comparative Fit Index) = 0.954 and RMSEA (Root Mean Square Error of Approximation) = 0.081, were considerably better (∆χ^2^ = 8111.9, ∆df = 6, *p* < 0.001) than that of the single-factor model (χ^2^ = 8926.9, SRMR = 0.259, CFI = 0.400, RMSEA = 0.286). Together, above two tests suggest that the common method bias is not serious in our dataset.

### 3.2. Preliminary Tests of Reliability, Validity, and Correlation

We performed preliminary analyses to test reliability, validity and correlation. First, in the reliability test, Cronbach α was used to measure the reliability of the scale. The results, as shown in Table 1, indicated that the Cronbach’s α coefficients of all measures ranged from 0.903 to 0.973, which are greater than the recommended threshold value of 0.70 [91], suggesting good internal consistency.

Second, a CFA was used to test two types of commonly reported construct validity, namely, convergent and discriminant validity. The convergent validity results presented in Table 1 show that all the standardized loadings were greater than 0.70 and that the average variance extraction (AVE) values of all constructs were greater than the expected cut-off value of 0.50 [92]. The composite reliability (CR) also exceeded the threshold of 0.70 [91]. Hence, these results provide evidence of a high convergent validity.

Third, the discriminant validity of the scale was tested by comparing the square root of AVE with the correlation coefficient between variables. The square root of AVE values should be higher than their inter-construct correlations to achieve discriminant validity. Table 2 reports the means, standard deviation, and correlation coefficients as well as the square root of AVE values. The results demonstrated a satisfactory discriminant validity of all constructs.

Lastly, as expected, results in Table 2 showed that two dimensions of safety-specific leader reward and punishment omission were negatively correlated with safety compliance behavior (*r*_SLRO_ = −0.148, *p* < 0.001; *r*_SLPO_ = −0.236, *p* < 0.001). Individual safety moral belief (*r* = 0.432, *p* < 0.001) and organizational size (*r* = 0.272, *p* < 0.05) were also found to be positively related to safety compliance behavior. Additionally, we found a negative relationship between gender and safety compliance behavior (*r* = −0.135, *p* < 0.001), suggesting that females are more likely to comply because of higher risk perception.

### 3.3. Hypothesis Testing

We conducted a multiple regression analysis to determine if there were significant main effects of SLRO and SLPO on safety compliance behavior, and the results are shown in Table 3. After accounting for the control variables, SLRO shows a slightly negative effect on safety compliance behavior (B = −0.099, *p* < 0.01; 95% CI: −0.168 to −0.03), while SLPO shows a highly negative effect on safety compliance behavior (B = −0.256, *p* < 0.001; 95% CI: −0.316 to −0.196), suggesting that Hypothesis 1 and Hypothesis 2 were supported.

To further confirm the differential effect between SLRO and SLPO on safety compliance behavior (H3), we utilized Mplus 7.0 to perform the bias-corrected bootstrapping method with 2000 iterations at a 95% confidence interval. The result suggests that the difference of effect size between SLRO and SLPO is significant (B = 0.157, *p* < 0.01; 95% CI: 0.064 to 0.254, BC CI excludes zero). Hence, this result lends support to Hypothesis 3, indicating that SLPO had a larger negative impact on SCB than SLRO.

**Figure 2:**
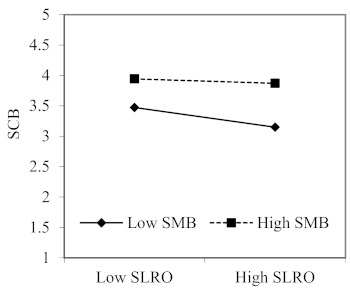
The interaction effect of SLRO and SMB on SCB. Note: SLRO, safety-specific leader reward omission, SLPO, safety-specific leader punishment omission, SMB, safety moral beliefs and SCB, safety compliance behavior.

**Figure 3:**
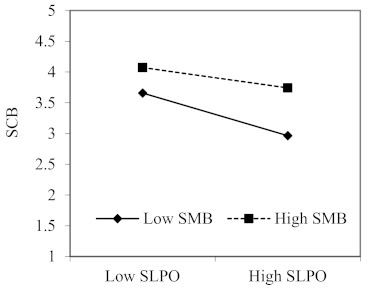
The interaction effect of SLPO and SMB on SCB. Note: SLRO, safety-specific leader reward omission, SLPO, safety-specific leader punishment omission, SMB, safety moral beliefs and SCB, safety compliance behavior.

We conducted a moderated regression analysis to determine if there were significant interactions between the hypothesized moderators and two predictors, and the results of moderating effects of safety moral belief and firm size are reported in Table 3. As expected, Hypothesis 4 and Hypothesis 5 were supported, because the interactions between SLRO and safety moral belief (B = 0.063, *p* < 0.05; 95% CI: 0.002 to 0.125), and between SLPO and safety moral belief (B = 0.091, *p* < 0.01; 95% CI: 0.029 to 0.153) have significant effects on the safety compliance behavior. To further interpret these interaction effects, following recommendations by Aiken and West [93], we plotted the two-way interactions using one standard deviation above and below the mean on SLRO (see Figure 2) and SLPO (see Figure 3). ln line with Hypothesis 4 and Hypothesis 5, the relationships between SLRO and safety compliance behavior, and between SLPO and safety compliance behavior were stronger among followers whose safety moral beliefs were low (1SD below the mean). Among followers with high safety moral beliefs (1SD above the mean), SLRO was virtually unrelated to SCB, while SLPO was slightly related to SCB.

**Figure 4:**
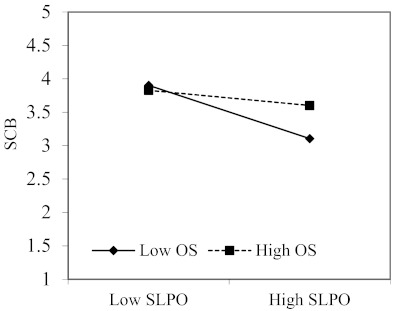
The interaction effect of SLPO and OS on SCB. Note: SLPO, safety-specific leader punishment omission, OS, organizational size and SCB, safety compliance behavior.

Consistent with H7, there is a significant interaction between SLPO and organizational size (B = 0.143, *p* < 0.001; 95% CI: 0.115 to 0.171) on safety compliance behavior. Unexpectedly, the interaction between SLRO and organizational size (H6) on safety compliance behavior was not statistically significant (*p* > 0.001; 95% CI includes zero). To further interpret the results, we also plotted the two-way interaction as Aiken and West [93] recommended (Figure 4). In support of H7, the negative relationship between SLPO and SCB is stronger when organizational size is small. High levels of safety compliance were seen if either SLPO was low or organizational size was large. Safety compliance was lowest when followers perceived a high level of SLPO and were working in small steel enterprises.

## 4. Discussion

Recently, interest in safety leadership as an antecedent of safety compliance has increased. The purpose of our study was to examine the relationships between safety-specific leader reward and punishment omission (a type of passive-avoidant leadership in the safety context) and followers’ safety compliance behaviors in the steel manufacturing context and how these associations may vary among steel workers at different levels of safety moral beliefs and organizational size. First, the results indicate that both safety-specific leader reward omission and punishment omission have good reliabilities and validities, suggesting that we succeed in contextualizing the general measures of leader reward and punishment omission [37] to the specific domain of occupational safety. Second, the findings show that both safety-specific leader reward omission and punishment omission are associated with followers’ safety compliance behaviors. Further, safety-specific leader punishment omission has a larger negative impact on followers’ safety compliance behaviors than safety-specific leader reward omission. Third, the results suggest that safety-specific leader reward omission (see Figure 2) and punishment omission (see Figure 3) have greater associations with followers’ safety compliance behavior when their safety moral belief is low than when it is high. Regarding the moderator of organizational size, the results indicate that safety-specific leader punishment omission has a greater relationship with followers’ safety compliance behavior in a small enterprise than in a large one, while such a moderating effect between safety-specific leader reward omission and organizational size on safety compliance behavior is not found (see Figure 4).

In addition to introducing the concept of leader reward and punishment omission [37], and contextually testing two measures in the occupational safety context, i.e., safety-specific leader reward omission and safety-specific leader punishment omission, the current study contributes to the safety and leadership research. First, expanding upon a significant body of the literature demonstrating the relationship between safety-specific passive-avoidant leadership and followers’ safety compliance behaviors [22,23,26,37], this study distinguishes between reward omission and punishment omission and further finds that they have differential effects on followers’ safety compliance behaviors where SLPO has a larger negative relationship with safety compliance behavior than SLRO, thus leading to a nuanced finding regarding the relationship between passive-avoidant leadership and followers’ safety compliance. Second, our study contributes largely to the existing body of knowledge by filling the leadership literature gap on a better understanding of the individual and organizational conditions under which passive-avoidant leadership influences safety compliance [46,48], with a particular emphasis on the moderating role played by safety moral belief of followers and organizational size in determining followers’ response to leaders’ nonreinforcement of their good or poor safety performance. This finding is, to some extent, in line with, Olsen, Hetland, Matthiesen, Hoprekstad, Espevik and Bakker [36], who demonstrated that individual moral disengagement changes the relationship between passive avoidant leadership and safety noncompliance and extends this stream of research by focusing on another moral belief of individual difference. More importantly, the findings highlight the buffering effect of organizational size. Researchers have argued that little is known about whether followers from large and small enterprises understand and respond differently to leaders’ avoidance of safety-specific rewards and punishments [45]. The present study offers prompt response to the call from Guo, Yiu and González [45] by demonstrating the differential effect of safety-specific leader punishment omission, and the same effect of safety-specific leader reward omission among steel workers from large and small enterprises. Third, although recent studies have examined the negative effect of passive-avoidant leadership on safety outcomes, this study offers evidence for the generalization of such effects with data from steel workers and, thus, replenish the safety-specific active-approaching leadership literature [18,19,94].

The results of the study have several practical implications. One of the implications is that leaders’ reward and punishment omission can decrease subordinates’ safety compliance behaviors. This suggests that if leaders want to motivate followers’ safety compliance behaviors, they should administer contingent reward or punishment rather than not respond to followers’ good or poor safety performance [57,95]. Further, although it is important for leaders to provide rewards, recognition and positive feedback contingently to followers based on their high safety performance and/or desired safety compliance behaviors, leaders should pay more attention to and make prompt punishments to followers’ poor safety performance and/or safety violation behaviors. Second, the moderating role of followers’ safety moral belief in the relationship of reward omission and punishment omission with safety compliance behavior indicates the significant benefits that may be brought through fostering moral belief related to workplace safety [61]. This means that organizations should provide safety-related training activities to persuade employees that safety violation is morally wrong and safety compliance is morally correct. Third, our findings suggest that managers in small and medium-sized enterprises should pay more attention to the crucial role of safety-specific punishments in ending employees’ safety violations, because they have greater motivation for safety violations due to poor working environment [8,43] and a lack of necessary safety equipment and climate [10,42,45].

Some methodological limitations in the present study must be acknowledged. First, we used a two-wave online survey to collect data and tried to exclude the alternative explanation of the passive-avoidant leadership-safety compliance link, which may decrease the chance of common method bias and draw causal conclusions [96]. Future studies can benefit from collecting data from multiple sources where supervisors may evaluate employees’ compliance behavior and followers evaluate supervisors’ reward and punishment omission and reexamining the causal connections by incorporating experimental or longitudinal design [96,97]. A second limitation is related to the representativeness of the present sample. The current survey was completed by front-line workers from steel enterprises with different organizational size in Anhui province with a response rate of 56.3%. Potential selection biases might have influenced the generalization of our findings. As such, more studies are recommended to replicate the present findings with more representative samples from more steel enterprises in other provinces in China and other countries, which may bolster the relevance of such findings to a broader audience. Third, social desirability, a limitation inherent in most research when respondents are asked to report items they look good or bad [98], might challenge our findings. Workplace safety compliance and individual safety moral belief are a sensitive issue for steel workers. Therefore, despite being told that the survey will be anonymous and confidential, respondents might still lie about actual safety compliance and moral belief due to a lack of credible assurance [98]. Future studies can benefit from particularly taking precautions to combat socially desirable responding such as rational analytic techniques and factor analytic techniques recommended by Mick and Glen [99].

## 5. Conclusions

Although employees in steel industry often perceive that leaders were absent and not responsive to their wishes or concerns regarding safety [8], little research has focused on such passive-avoidant leadership behavior, and no empirical research has examined safety-specific passive-avoidant leadership in steel industry [2]. To fill this gap, this study explored two dimensionalities of safety-specific leader reward and punishment omission and how these dimensions related to followers’ safety compliance behaviors and further examined whether the relationship is moderated by safety moral beliefs and organizational size in a sample of 704 steel workers in China. We substantiated that the two safety-specific leadership styles, i.e., reward omission and punishment omission, will decrease followers’ safety compliance behaviors, and these effects will be attenuated by safety moral beliefs and organizational size. Our results suggest that theory and practice would benefit from recognizing and embracing that supervisors’ omissions and avoidances of good and poor safety performance have significant effects on followers’ safety compliance behavior and that informal safety moral beliefs and formal reward and punishment policies are required to prevent and reduce the effect of such counterproductive and non-considerate leader behaviors on safety compliance.

## Figures and Tables

**Table 1:** Scale items and results of CFA.

Scale Items	Std. Loadings	AVE	CR
*Safety-specific Leader Reward Omission [Adapted from Hinkin &Schriesheim (2008)] Cronbach’s α = 0.911*			
I often do my jobs safely and still receive no praise from my manager.	0.732	0.634	0.912
My manager often gives me no feedback when I do my jobs safely.	0.761		
When I do my jobs safely my manager usually does nothing.	0.793		
My safety performance often goes unacknowledged by my manager.	0.746		
I don’t often get praised by my manager when I perform safely.	0.871		
My safety performance often gets no response from my manager.	0.864		
*Safety-specific Leader Punishment Omission [Adapted from Hinkin& Schriesheim (2008)] Cronbach’s α = 0.973*			
I seldom get criticized by my manager when I perform unsafely.	0.892	0.880	0.973
My manager gives me no feedback when I perform unsafely.	0.968		
When I perform unsafely in my job I receive no criticism from my manager.	0.896		
When I perform unsafely my manager does nothing.	0.967		
My unsafety performance often gets no response from my manager.	0.963		
*Safety Compliance Behavior [Neal & Griffin (2006) and Mei et al. (2017)] Cronbach’s α = 0.963*			
I used all the necessary safety equipment to do my job in past week.	0.830	0.846	0.965
I used the correct safety procedures for carrying out my job in past week.	0.911		
I ensured the highest levels of safety when I carry out my job in past week.	0.956		
I strictly abided by workplace safety policies when I carry out my job in past week.	0.951		
I performed safety duties and obligations when I carry out my job in past week.	0.945		
*Safety Moral Beliefs [Adapted from Vance & Siponen (2012)] Cronbach’s α = 0.903*			
I feel that it would be wrong to violate company safety production rules and operation procedures.	0.881	0.764	0.907
It is morally right to violate company safety production rules and operation procedures (R).	0.898		
How morally wrong would it be if employees violate company safety production rules and operation procedures?	0.843		

Note: AVE, average variance extraction; CR, composite reliability.

**Table 2:** Means, SD, correlation coefficients of all variables and square root of AVE values of all constructs.

Variables	Mean	SD	GEN	AGE	EDU	INC	EMF	WY	OS	SLRO	SLPO	SMB	SCB
GEN	1.382	0.486											
AGE	3.097	0.903	−0.081 *										
EDU	2.173	0.832	−0.101 **	−0.422 ***									
INC	2.088	0.940	−0.254 ***	0.013	0.232 ***								
EMF	1.308	0.848	0.107 **	0.026	−0.104 **	−0.116 **							
WY	4.335	1.796	−0.072	0.452 ***	−0.080 *	0.178 ***	−0.142 ***						
OS	3.929	1.400	0.034	−0.099 **	−0.037	−0.151 ***	0.036	−0.020					
SLRO	1.892	0.427	0.112 **	0.005	−0.023	−0.065	−0.071	−0.001	0.134 ***	**0.796**			
SLPO	1.605	0.482	0.124 **	0.049	−0.107 **	−0.204 ***	−0.110 **	0.031	0.322 ***	0.369 ***	**0.938**		
SMB	4.877	0.379	−0.062	0.080 *	−0.036	−0.036	0.022	0.000	0.083 *	−0.018	−0.080 *	**0.874**	
SCB	4.794	0.393	−0.135 ***	0.034	−0.010	−0.065	0.011	0.029	0.272 ***	−0.148 ***	−0.236 ***	0.432 ***	**0.920**

Note: GEN, gender: Men = 1, Women = 2; AGE, age: 20–30, 31–40, 41–50, ˃50; EDU, educational level: Junior high school or below, High school or technical secondary school, University or senior college, Master or above; INC, monthly income: ˂3000, 3000–5000, 5001–7000, 7001–10,000 and ˃10,000; EMF, employment: Formal = 1, Informal = 2; WY, working years: ˂1, 1–2, 3–4, 5–6, 7–10, 11–15 and ˃15; OS, organizational size: ˂50, 50–300, 301–500, 501–1000, 1001–5000 and ˃5000; SLRO, safety-specific leader reward omission; SLPO, safety-specific leader punishment omission; SMB, safety moral beliefs; SCB, safety compliance behavior. The square root of AVE values are bold and reported in diagonal. *** *p* < 0.001, ** *p* < 0.01 and * *p* < 0.05.

**Table 3:** Multiple and moderated regression results for the main effect and moderating effect (*N* = 704).

Variable	B	SE	*t*	Sig.	95% CI	
					Lower	Upper
Constant	3.609	0.190	18.984	0.000	3.236	3.983
*Control variables*						
GEN	−0.086	0.024	−3.612	0.000	−0.133	−0.039
AGE	0.005	0.015	0.350	0.727	−0.025	0.036
EDU	0.008	0.015	0.517	0.605	−0.022	0.038
INC	−0.038	0.013	−2.978	0.003	−0.064	−0.013
EMF	−0.003	0.014	−0.229	0.819	−0.030	0.023
WY	0.012	0.007	1.691	0.091	−0.002	0.026
*Independents*						
SLRO	−0.099	0.035	−2.816	0.005	−0.168	−0.030
SLPO	−0.256	0.031	−8.360	0.000	−0.316	−0.196
*Moderators*						
SMB	0.298	0.032	9.172	0.000	0.234	0.362
OS	0.105	0.009	12.256	0.000	0.088	0.122
*Interactions*						
SLRO*SMB	0.063	0.031	2.020	0.044	0.002	0.125
SLPO*SMB	0.091	0.032	2.875	0.004	0.029	0.153
SLRO*OS	−0.017	0.012	−1.414	0.158	−0.040	0.007
SLPO*OS	0.143	0.014	10.125	0.000	0.115	0.171

Note: GEN, gender; AGE, age; EDU, educational level; INC, monthly income; EMF, employment; WKY, working years; OS, organizational size; SLRO, safety-specific leader reward omission; SLPO, safety-specific leader punishment omission; SMB, safety moral beliefs; and SCB, safety compliance behavior. The results of multiple and moderated regression analysis were reported in the same table.

## Data Availability

The dataset used in this research are available upon request from the corresponding author. The data are not publicly available due to restrictions i.e., privacy or ethical.
